# Supplementary material for: A fault-intrusion-tolerant system and deadline-aware algorithm for scheduling scientific workflow in the cloud
Source: PeerJ Comput Sci. 2021 Nov 2;7:e747. doi: 10.7717/peerj-cs.747 (PMC8576554; doi:10.7717/peerj-cs.747)
Supplement: Supplemental Information 6 [file peerj-cs-07-747-s006.zip › Myproject1.1/docs/org/cloudbus/cloudsim/HostStateHistoryEntry.html]

HostStateHistoryEntry


JavaScript is disabled on your browser.


- Overview
- Package
- Class
- Use
- Tree
- Deprecated
- Index
- Help

- Prev Class
- Next Class

- Frames
- No Frames

- All Classes

- Summary:
- Nested |
- Field |
- Constr |
- Method

- Detail:
- Field |
- Constr |
- Method


org.cloudbus.cloudsim

## Class HostStateHistoryEntry

- java.lang.Object
- - org.cloudbus.cloudsim.HostStateHistoryEntry

- ---

    

  ```
  public class HostStateHistoryEntry
  extends java.lang.Object
  ```

  The Class HostStateHistoryEntry.

  Since:
  :   CloudSim Toolkit 2.1.2

- - ### Constructor Summary

    Constructors

    Constructor and Description

    `HostStateHistoryEntry(double time,
    double allocatedMips,
    double requestedMips,
    boolean isActive)`

    Instantiates a new vm mips allocation history entry.
  - ### Method Summary

    Methods

    | Modifier and Type | Method and Description |
    | --- | --- |
    | `double` | `getAllocatedMips()` Gets the allocated mips. |
    | `double` | `getRequestedMips()` Gets the requested mips. |
    | `double` | `getTime()` Gets the time. |
    | `boolean` | `isActive()` Checks if is active. |
    | `void` | `setActive(boolean isActive)` Sets the active. |
    | `protected void` | `setAllocatedMips(double allocatedMips)` Sets the allocated mips. |
    | `protected void` | `setRequestedMips(double requestedMips)` Sets the requested mips. |
    | `protected void` | `setTime(double time)` Sets the time. |

    - ### Methods inherited from class java.lang.Object

      `clone, equals, finalize, getClass, hashCode, notify, notifyAll, toString, wait, wait, wait`

- - ### Constructor Detail


    - #### HostStateHistoryEntry

      ```
      public HostStateHistoryEntry(double time,
                           double allocatedMips,
                           double requestedMips,
                           boolean isActive)
      ```

      Instantiates a new vm mips allocation history entry.

      Parameters:
      :   `time` - the time
      :   `allocatedMips` - the allocated mips
      :   `requestedMips` - the requested mips
      :   `isActive` - the is active
  - ### Method Detail


    - #### setTime

      ```
      protected void setTime(double time)
      ```

      Sets the time.

      Parameters:
      :   `time` - the new time


    - #### getTime

      ```
      public double getTime()
      ```

      Gets the time.

      Returns:
      :   the time


    - #### setAllocatedMips

      ```
      protected void setAllocatedMips(double allocatedMips)
      ```

      Sets the allocated mips.

      Parameters:
      :   `allocatedMips` - the new allocated mips


    - #### getAllocatedMips

      ```
      public double getAllocatedMips()
      ```

      Gets the allocated mips.

      Returns:
      :   the allocated mips


    - #### setRequestedMips

      ```
      protected void setRequestedMips(double requestedMips)
      ```

      Sets the requested mips.

      Parameters:
      :   `requestedMips` - the new requested mips


    - #### getRequestedMips

      ```
      public double getRequestedMips()
      ```

      Gets the requested mips.

      Returns:
      :   the requested mips


    - #### setActive

      ```
      public void setActive(boolean isActive)
      ```

      Sets the active.

      Parameters:
      :   `isActive` - the new active


    - #### isActive

      ```
      public boolean isActive()
      ```

      Checks if is active.

      Returns:
      :   true, if is active


- Overview
- Package
- Class
- Use
- Tree
- Deprecated
- Index
- Help

- Prev Class
- Next Class

- Frames
- No Frames

- All Classes

- Summary:
- Nested |
- Field |
- Constr |
- Method

- Detail:
- Field |
- Constr |
- Method
